# Impact of COVID-19 on clinical outcomes and care delivery in pediatric oncology patients in Lebanon in 2020–2023: a retrospective study

**DOI:** 10.3389/fped.2025.1608740

**Published:** 2025-07-09

**Authors:** Peter Noun, May Farhat, Hassan Khalife, Jennifer Bou Younis, Mohamad Farhat, Hadi Kabbout, Hammam Haridy, Jihane Moussa, Florence Lefebvre d’Hellencourt, Srinivas Rao Valluri, Julia Spinardi, Nadine Al Akoury, Moe H. Kyaw, Adlette Inati

**Affiliations:** ^1^ Department of Pediatrics, Hematology Oncology Unit, Saint George Hospital University Medical Center, Beirut, Lebanon; ^2^ Saint George University, School of Medicine, Beirut, Lebanon; ^3^ Department of Epidemiology and Population Health, Faculty of Health Sciences, American University of Beirut, Beirut, Lebanon; ^4^ Department of Pediatrics Hematology Oncology, Sahel Hospital Medical Center (Lebanese University), Beirut, Lebanon; ^5^ Department of Pediatrics, Rai Hospital, Saida, Lebanon; ^6^ Department of Pediatrics, Hematology Oncology Lebanese University Hospital Geitewi, Beirut, Lebanon; ^7^ Department of Pediatrics, Hematology Oncology CHU Notre Dame Des Secours, Byblos, Lebanon; ^8^ Scientific and Medical Affairs, Emerging Markets, Pfizer Inc., Collegeville, PA, United States; ^9^ Department of Pediatrics, LAU Gilbert and Rose-Marie Chaghoury School of Medicine, Byblos, Lebanon; ^10^ Division of Pediatric Hematology Oncology, NINI Hospital, Tripoli, Lebanon

**Keywords:** COVID-19, oncology, pediatric, children, epidemiology, management, diagnosis, care

## Abstract

**Introduction:**

The COVID-19 pandemic has significantly impacted the pediatric population worldwide, particularly those with comorbidities who often experience more severe outcomes. However, the impact of COVID-19 on pediatric oncology patients remain poorly understood.

**Methods:**

This retrospective observational study examined data from 85 pediatric oncology patients aged <18 years who tested positive for COVID-19 at five study sites across Lebanon from March 2020 to May 2023. Detailed demographic, clinical, treatment, healthcare resource utilization (HCRU), and disease outcomes were collected. Outcomes was summarized descriptively and two-sided 95% confidence intervals (CI) were calculated using the Clopper-Pearson method.

**Results:**

There was 85 pediatric oncology patients diagnosed with COVID-19, with a mean age of 11.57 years. Of these, 72 patients (84.6%) had hematological malignancies and 13 (15.4%) had solid tumors. Thirteen patients (15.3%) had comorbidities. Treatment delays on oncology care occurred in 61 patients (71.8%). Twelve patients (14.1%) required hospitalization, four of whom needed intensive care unit (ICU) admission, with ICU stays ranging from 1 day to 2 months. COVID-19 related mortality was 4.7%.

**Discussion:**

COVID-19 was associated with changes or delay in oncological treatment. In addition, the burden of COVID related hospitalization, intensive care utilization and death were substantial among pediatric cancer patients. Our findings highlight the importance of vaccination in pediatric oncology patients.

## 1 Introduction

The outbreak of Coronavirus Disease 2019 (COVID-19), caused by the novel severe acute respiratory syndrome coronavirus 2 (SARS-CoV-2), rapidly evolved into a global health pandemic, as officially declared by the World Health Organization (WHO) (1). The pandemic particularly captured the interest of pediatric oncologists, who closely monitored its impact on children with cancer.

Adult cancer patients with COVID-19 experienced severe illness, with significantly higher mortality rates compared to other age groups (2–5). Many case reports and cohort studies from national and international registries have shown that the majority of pediatric cancer patients experience only clinically mild to moderate symptoms (6–11). In fact, only a small percentage of them progress to severe disease and necessitate critical care (12).

Despite the generally milder impact of COVID-19 on children, the pandemic has introduced numerous challenges to the healthcare sector, particularly affecting the overall management of pediatric cancer patients. These challenges include significant delays in the diagnosis of cancer due to disruptions in routine healthcare services, interruptions in necessary hospitalizations, and major disruptions to ongoing cancer treatments (13–15). The pandemic has also complicated treatment administration and follow-up care, necessitating adjustments in clinical practice to ensure continued care while minimizing exposure to the virus (14, 16). Moreover, families of children with cancer faced a decline in income due to the pandemic, which adversely affected their ability to access care and resources. Finally, the migration crisis, in addition to the economic crisis and sociopolitical instability in Lebanon since 2019 have further exacerbated the challenges for the healthcare sector in general, and the situation for pediatric cancer patients specifically, due to critical shortages of essential resources. This includes a severe lack of medications that are vital for the treatment of the pediatric oncology population (17, 18).

Data on the severity of COVID-19 in pediatric patients with cancer in the Middle East is limited and not well understood. To address this knowledge gap, we hereby describe the impact of COVID-19 on the clinical outcomes and overall care delivery of pediatric oncology patients (≤18 years of age) who tested positive for COVID-19 in Lebanon between March 2020 and May 2023. We also outline the specific challenges that were faced by these patients during the pandemic and the additional support or resources required to manage their care effectively.

## 2 Materials and methods

This retrospective, observational database study included all pediatric patients aged 0–18 years diagnosed with any type of cancer, including both hematologic malignancies and solid tumors, and who tested positive for SARS-CoV-2 between March 2020 and May 2023 during any visit at one of the five participating hospitals and referral centers in Lebanon. These included: Saint George Hospital University Medical Center in Achrafieh, Beirut (Mid Lebanon); NINI Hospital in Tripoli (North Lebanon); Notre Dame de Secours Hospital in Jbeil (Mount Lebanon); Lebanese Hospital Geitaoui in Achrafieh, Beirut (Mid Lebanon) and Al Raii Hospital in Sidon (South Lebanon). Collectively, these five centers account for approximately 40%–45% of all pediatric oncology patients in Lebanon. The study data were extracted from patient medical records and hospital databases for further analyses (*N* = 210). Patients were categorized based on their COVID-19 status, with two distinct groups: 85 patients who tested positive for COVID-19 and 125 patients who tested negative by PCR. Exclusion criteria included any patient who received a COVID-19 vaccine prior to their SARS-CoV-2 infection. Institutional Review Board (IRB) approval for this study was obtained from all participating centers. As this was an observational, non-interventional study with anonymized data, patients' caregiver consent was not needed.

The primary endpoint of the study was to determine the proportion of pediatric oncology patients, categorized by specific demographics, clinical, and hematologic characteristics, who experienced changes in their outcomes and therapy due to COVID-19 infection. The secondary endpoints of the study included several key measures. First, the proportion of pediatric oncology patients who experienced delays or interruptions in their treatment course, based on pre-established criteria, both with and without COVID-19 infection. A delay was defined as a postponement of seven or more days from the originally scheduled date of treatment. “Treatment delay” encompasses all types of oncologic therapies, including chemotherapy, radiotherapy, surgery, and scheduled imaging. Second, the proportion of these patients who were hospitalized specifically due to COVID-19 were assessed. Third, the study evaluated the rate of COVID-19 re-infections among pediatric oncology patients. Fourth, it examined the proportion of COVID-19 positive pediatric oncology patients who demonstrated pre-defined measures of disease progression and oncologic severity related to their COVID-19 status. Fifth, the incidence of thrombosis among COVID-19 positive pediatric oncology patients was also analyzed. Finally, the study assessed the healthcare resource utilization (HCRU) related to the sequelae of COVID-19 among this patient population.

All statistical analyses were conducted using Stata 18. The primary analysis used descriptive statistics to summarize the clinical and demographic characteristics of pediatric oncology patients, categorized by their COVID-19 status (positive or negative). Continuous variables, such as age and hematological measurements (e.g., hemoglobin levels), were reported as means with standard deviations (mean ± SD). Categorical variables, including gender, cancer type, and comorbidities, were presented as counts and percentages. In order to estimate the precision of these percentages, two-sided 95% confidence intervals (CIs) for the point estimates were calculated using the Clopper-Pearson exact method. Missing data were addressed by including missing values in the analysis without imputation. The presence of missing values was also noted and reported in the relevant tables.

Comparative analyses were conducted to assess differences in demographic and clinical characteristics between COVID-19 positive and negative pediatric cancer patients. Categorical variables were compared using the Chi-square test when expected cell counts were ≥5, and the Fisher's exact test was applied for 2 × 2 tables with small cell sizes. For continuous variables (white blood cell count, hemoglobin level, platelet count,…), independent samples *t*-tests with unequal variances (Welch's *t*-test) were employed. A two-sided *p*-value <0.05 was considered indicative of statistical significance.

## 3 Results

During the study period, we monitored a total of 210 pediatric oncology patients undergoing active chemotherapy. Among these, we identified 85 (40.5%) patients who tested positive for COVID-19 through RT-PCR. This cohort included 48 (56.5%) males and 37 (43.5%) females, with a mean age of 11.6 + 4.4 years. More details on the demographic characteristics of the pediatric oncology children across all five centers are presented in Table 1.

**Table 1:** Patient demographics and characteristics.

Characteristics	Overall % (*N*)	COVID-19 positive % (n)	COVID-19 negative %(n)
Pediatric oncology patients	100% (210)	40.5% (85)	59.5% (125)
Age in years (mean ± SD)	11.12 ± 5.1	11.57 ± 4.4	10.79 ± 5.5
Gender			
Male	54.3% (114)	56.5% (48)	52.8% (66)
Female	45.7% (96)	43.5% (37)	47.2% (59)
Treating Center			
Saint George UH	37.6% (79)	32.9% (28)	40.8% (51)
Geitaoui	29.5% (62)	20.0% (17)	36.0% (45)
Notre Dame de Secours	14.3% (30)	16.5% (14)	12.8% (16)
NINI Hospital	13.8% (29)	24.7% (21)	6.4% (8)
Al Raii Hospital	4.8% (10)	5.9% (5)	4.0% (5)

A total of 72 COVID patients (84.7%) had a hematological malignancy, while 13 (15.3%) had solid tumors (Table 2). At the time of COVID-19 diagnosis, 69 patients (81.2%) were receiving chemotherapy, 1 patient (1.2%) was receiving immunotherapy, and the remaining 15 patients (17.6%) were on a combined regimen. Of the COVID-19 positive patients, 13 patients (15.3%) had comorbidities, which included respiratory, genetic, endocrine, metabolic, and neurological among other comorbidities (Table 2). While individual comorbidity types were not significantly different between groups, the overall absence of comorbidities was significantly more common among COVID-negative patients (*p* = 0.002). When compared to their COVID-19 negative counterparts, we observed that COVID-19 positive patients had lower white blood count (WBC) 9.3 × 10^4^ ± 1.9 × 10^4^ and platelet count (8.9 × 10^4^ ± 10.5 × 10^4^) and higher erythrocyte sedimentation rate (ESR) levels (41.3 ± 26.8). WBC count (*p* = 0.049) and platelet count (*p* = 0.014) were significantly different between groups, whereas hemoglobin (*p* = 0.288) and ESR (*p* = 0.704) were not, implicating that COVID-19 may have a notable impact on hematological parameters in pediatric oncology patients. Among the 85 patients who were in the midst of anticancer therapy, 61 patients (71.8%) experienced modifications or delay in their treatment as a result of COVID-19 infection (Table 2). This delay was significantly more frequent among COVID-positive patients compared to COVID-negative ones (*p* < 0.001).

**Table 2:** Primary endpoints of cancer and COVID-19 outcomes.

Clinical and treatment characteristics	Overall % (*N*)	COVID-19 positive % (n)	95% CI [%]	COVID-19 negative % (n)	95% CI [%]	*P*-value
Cancer type						0.218
Hematological	**80% (168)**	**84.7% (72)**	**[75.6, 90.8]**	**76.8% (96)**	**[68.7, 83.3]**	
Low	15.2% (32)	5.9% (5)	[1.9, 13.2]	21.7% (27)	[14.9, 30.1]	
Intermediate	13.3% (28)	17.6% (15)	[10.2, 27.4]	10.5% (13)	[5.7, 17.3]	
High risk	51.5% (107)	61.2% (52)	[60, 0.82]	44.3% (55)	[35.4, 53.4]	
*Missing*	*1*	*0*	–	*1*		
Solid	**20% (42)**	**15.3% (13)**	**[9.2, 24.4]**	**23.2% (29)**	**[16.1, 31.6]**	
Stage 1	1.4% (3)	1.1% (1)	[0.03, 6.4]	2.4% (3)	[0.5, 6.8]	
Stage 2	4.3% (9)	5.9% (5)	[1.9, 13.2]	3.2% (4)	[0.9, 8.1]	
Stage 3	8.6% (18)	5.9% (5)	[1.9, 13.2]	10.5% (13)	[5.7, 17.3]	
Stage 4	5.7% (12)	2.4% (2)	[0.3, 8.2]	7.3% (9)	[3.4, 13.3]	
Treatment						0.441
Chemotherapy	80.4% (168)	81.2% (69)	[71.2, 88.8]	79.8% (99)	[71.7, 86.5]	
Immunotherapy	0.9% (2)	1.2% (1)	[0.03, 6.4]	0% (0)	[0, 2.91]	
Combined*	18.7% (39)	17.6% (15)	[10.2, 27.4]	20.2% (25)	[13.5, 28.3]	
*Missing*	*1*	*0*		*1*		
Treatment Duration						0.090*
< 1 year	18.6% (39)	12.9% (11)	[6.6, 21.9]	22.6% (28)	[15.6, 30.9]	
1–2 years	28.7% (60)	34.1% (29)	[24.2, 45.2]	25.0% (31)	[17.7, 33.6]	
>2–3.5	39.2% (82)	35.3% (30)	[25.2, 46.4]	41.9% (52)	[33.1, 51.1]	
>3.5–6 years	11.5% (24)	16.5% (14)	[9.3, 26.1]	8.1% (10)	[3.9, 14.3]	
>6 years	2.0% (4)	1.2% (1)	[0.03, 6.4]	2.4% (3)	[0.5, 6.9]	
*Missing*	*1*	*0*		*1*		
Delay in treatment						0.000*
Yes	30.5% (64)	71.8% (61)	[60.9, 81.0]	2.4% (3)	**[0.5, 6.9]**	
No	69.5% (146)	28.2% (24)	[19.0, 39.0]	97.6% (122)	**[93.1, 99.5]**	
Comorbidity						
Respiratory	1.5% (3)	3.5% (3)	[0.7, 9.9]	0% (0)	[0, 2.91]	0.065
Genetic	1.5% (3)	2.4% (2)	[0.3, 8.2]	0.8% (1)	[0.02, 4.4]	0.567
Endocrine & Metabolic	2.4% (5)	4.7% (4)	[1.3, 11.6]	0.8% (1)	[0.02, 4.4]	0.160
Neurological	0.9% (2)	2.4% (2)	[0.3, 8.2]	0% (0)	[0, 2.91]	0.163
Other	0.9% (2)	2.4% (2)	[0.3, 8.2]	0% (0)	[0, 2.91]	0.163
None	92.8% (195)	84.7% (72)	[75.3, 91.6]	98.4% (123)	[94.3, 99.8]	0.002*
Hematological levels (mean ± SD)						
WBC at Dx (/μl)	1.6 × 10^4^ ± 4.4 × 10^4^	9.3 × 10^4^ ± 1.9 × 10^4^		1.9 × 10^4^ ± 5.5 × 10^4^		0.049*
Hb at Dx (g/dl)	9.72 ± 2.6	9.9 ± 3.2		9.6 ± 2.1		0.288
Platelet count at Dx (/μl)	11.2 × 10^4^ ± 11.7 × 10^4^	8.9 × 10^4^ ± 10.5 × 10^4^		12.8 × 10^4^ ± 12.2 × 10^4^		0.014
ESR at Dx (mm/h)	40.5 ± 25.4	41.3 ± 26.8		39.9 ± 24.5		0.704

Indicated in bold are the percentage and total number of patients per cancer type, i.e. hematological and solid tumors.

* Indicates statistical significance at *p* < 0.05.

In terms of COVID-19 signs and symptoms, the majority of the patients (92.9%) were symptomatic but had mild disease symptoms (82.3%). Of these patients, the most common symptoms were systemic (fever, chills, tiredness/weakness) (79.7%), respiratory symptoms (54.4%), and neurologic symptoms (headaches) (40.5%). Notably, two patients developed thrombosis in the form of pulmonary embolism and received low-molecular-weight heparin (LMWH) (Table 3). All patients received appropriate treatment for the management of their COVID-19-related symptoms, including antibiotic and steroid therapies. None of the children received hydroxychloroquine, remdesivir, favipiravir, or tocilizumab.

**Table 3:** Severity of COVID-19 in pediatric cancer patients (*n* = 85).

Characteristics	COVID-19 positive % (n)	95% CI [%]
Treatment Stage at Time of COVID-19		
Induction/Course/Cycle	18.9% (14)	[10.7, 29.7]
Consolidation	6.8% (5)	[2.2, 15.1]
Re-intensification	8.1% (6)	[3.0, 16.8]
Maintenance	66.2% (49)	[54.2, 76.8]
*Missing*	*11*	
COVID-19 Re-infection		
Yes	3.5% (3)	[0.7, 9.9]
No	96.5% (82)	[90.0, 99.3]
Symptomatic		
Yes	92.9% (79)	[85.3, 97.3]
No	7.1% (6)	[2.6, 14.7]
Severity Index (*n* = 79)		
Mild	82.3% (65)	[72.1, 89.9]
Moderate	11.4% (9)	[5.3, 20.5]
Severe	6.3% (5)	[2.1, 14.2]
Specific Symptoms (*n* = 79)*		
Respiratory	54.4% (43)	[42.8, 65.7]
Gastrointestinal	16.5% (13)	[9.1, 26.5]
Systemic	79.7% (63)	[69.6, 87.1]
Neurologic	40.5% (32)	[30.4, 51.5]
Dermatological	1.2% (1)	[0.03, 6.9]
Thrombosis		
Yes	2.4% (2)	[0.3, 8.2]
No	97.6% (83)	[91.8, 99.7]

* Patients may have more than one symptom.

Out of the COVID-positive cohort, a total of 12 (14.1%) patients required hospital admission due to COVID-19 for a duration ranging from short stays of 0–15 days (10.6%, *n* = 9), 15–30 days (2.3%, *n* = 2), and up to three months (1.2%, *n* = 1). Among these cases, four (1.9%) patients necessitated admission to the intensive care unit (ICU), with lengths of stay ranging anywhere from one day to two months (Table 4). The overall mortality rate among patients who tested positive for COVID-19 was 17.7%. Of these, 11 deaths were attributed to progression of oncologic disease, while four were due to COVID-19. However, the difference in mortality between COVID-positive and negative groups was not statistically significant (*p* = 0.086), nor was the difference in cause of death due to cancer progression or COVID-19 (*p* = 0.365). Notably, all four COVID-19-related deaths occurred in patients with refractory/progressive disease or relapse. Moreover, three patients (3.5%) tested positive again for COVID-19, defined as a new positive PCR test following documented resolution of their initial infection at the beginning of the study (Table 4).

**Table 4:** Healthcare resource utilization and COVID-19 reinfection rates.

Characteristics	Overall % (*N*)	COVID-19 positive % (n)	95% CI [%]	COVID-19 negative % (n)	95% CI [%]	*P*-value
COVID-19 care setting						
Hospitalization	5.7% (12)	14.1% (12)	[7.5, 23.4]	0% (0)	[0, 0.03]	—
Clinic Visits	94.3% (198)	85.9% (73)	[76.6, 92.5]	100% (125)	[97.1, 100]	0.000*
Duration of hospitalization						—
0–15 days	4.3% (9)	10.6% (9)	[4.9, 19.1]	0% (0)	[0, 2.91]	
15–30 days	0.5% (2)	2.3% (2)	[0.3, 8.2]	0% (0)	[0, 2.91]	
3 months	0.5% (1)	1.2% (1)	[0.03, 6.4]	0% (0)	[0, 2.91]	
ICU Need						0.026*
Yes	1.9% (4)	4.7% (4)	[1.3, 11.6]	0% (0)	[0, 2.91]	
No	98.1% (206)	9.4% (8)	[4.1, 17.7]	0% (0)	[0, 2.91]	
Duration of ICU stay						—
0–15 days	0.95% (2)	2.3% (2)	[0.3, 8.2]	0% (0)	[0, 2.91]	
15–30 days	0.47% (1)	1.2% (1)	[0.03, 6.4]	0% (0)	[0, 2.91]	
2 months	0.47% (1)	1.2% (1)	[0.03, 6.4]	0% (0)	[0, 2.91]	
COVID-19 Re-infection						—
Yes	1.4% (3)	3.5% (3)	[0.7, 9.9]	0% (0)	[0, 2.91]	
No	207 (207)	96.5% (82)	[90.0, 99.3]	100% (125)	[97.1, 100]	
Death						0.086
Yes	12.38% (26)	17.7% (15)	[10.2, 27.4]	8.8% (11)	[4.5, 15.2]	
No	87.62% (184)	82.3% (70)	[72.6, 89.8]	91.2% (114)	[84.8, 95.5]	
Cause of death						
Oncologic disease progression	10.5% (22)	12.9% (11)	[7.4, 21.7]	8.8% (11)	[4.5, 15.2]	0.365
COVID-19	1.9% (4)	4.7% (4)	[1.3, 11.6]	0% (0)	[0, 2.91]	**—**

* Indicates statistical significance at *p* < 0.05.

## 4 Discussion

The present study demonstrates considerable burden of morbidity and mortality rates, treatment delays, and need for hospitalization and intensive care among pediatric cancer patients with COVID-19 in Lebanon. Our findings also highlight that COVID-19 infection may have had a notable impact on hematological parameters in pediatric oncology patients and contributed to higher hospitalization and ICU admission with longer stay in children with oncologic conditions. These data underscore the importance of effective preventive measure in this at-risk population.

In our study, 61 (71.8%) COVID patients had their treatment delayed suggesting a substantial impact of COVID-19 pandemic on healthcare system and practice related to the management of pediatric oncology patients. Balancing the interruption of cancer treatment against the risk of malignancy progression can be challenging, especially in newly diagnosed patients (19, 20). However, a small case series by Kakunje et al. reported no delays in chemotherapy treatment among children with cancer diagnosed with COVID-19 (21). Additional large studies are needed to quantify the true impact of COVID-19 on treatment delay and disruption of healthcare services not only during this past 4 years but also in the years to come.

Comorbidities in pediatric cancer patients with COVID-19 may further complicate their clinical management and overall prognosis. Toluney et al. reported that four patients had comorbidities unrelated to COVID-19 in addition to their cancer diagnosis; however, no mortality was observed in these patients. Similarly, in our study, 13 (15%) pediatric oncology patients who tested positive for COVID-19 had various comorbidities, including respiratory, genetic, endocrine, metabolic, neurological among others. Notably, 11 patients passed away due to comorbidities unrelated to COVID-19, primarily as a result of disease progression.

Data from other studies have shown that the most common COVID-19 symptoms observed in pediatric patients were cough and fever, a finding consistent with studies involving cancer patients (21–26). In our study, fever and cough were also among the predominant symptoms. When evaluating the severity of COVID-19 among pediatric oncology patients, we found that most children were symptomatic but experienced mild forms of the disease. This is consistent with other reports from the literature (24, 25).

The mortality rate of 4.7% (four deaths) reported in the present study within the COVID-19 patients is comparable to other studies of COVID-19 infection in pediatric oncology patients (27–30). All four dead patients had refractory/progressive disease or were in relapse at the time of COVID-19 infection. One study by Parker et al. and de Rojas et al. reported no COVID-19 deaths (19). However, the mortality rate from COVID-19 was found to be 28% in a study by Arous et al. and 4.4% in a study by Tolunay et al. (26, 31). Hospitalization rates among pediatric oncology patients with COVID-19 infection have been a growing concern in recent studies. Parker et al. found a significant rate of COVID-19-related hospitalizations, with more than one in four patients and over one in three symptomatic patients requiring admission (19). Similarly, in our study we also had 12 (5.7%) cases of hospitalization. The need for ICU admission was also reported by several studies. Antúnez-Montes et al. noted that among children with immunodeficiency, the need for ICU rose to 11.5%, despite the overall rate of immunodeficient children being only 4.4% (32). Likewise, among patients with a history of chemotherapy, the ICU requirement was 7.7%, compared to an overall rate of 3.4% (32). Moreover, in a study by Toluney et al., the rate of ICU admission was reported at 8.9% among pediatric cancer patients (26). In our cohort of pediatric oncology patients who tested positive for COVID-19, the rate of ICU admission was found to be 4.7%.

Reinfection of pediatric oncology patients with COVID-19 raises important concerns for both clinical management and patient outcomes. In our study, three (3.5%) patients tested positive for COVID-19 for a second time. Similarly, Hashmi et al. reported a higher rate of COVID reinfection in 11/110 (10%) patients with hematological malignancy (20).

While initial infections can lead to a range of symptoms, reinfections may present differently, potentially resulting in varying degrees of severity (sometimes more severe in nature) and new challenges. Given the immunocompromised status of many pediatric oncology patients due to their underlying conditions and treatments, they may be at higher risk for both initial infections and subsequent reinfections, particularly with the rise of new COVID-19 strains and variants (20, 33). While this study provides valuable insights, several limitations should be acknowledged. First, the retrospective nature of the study may introduce biases that could impact the findings. Additionally, the heterogeneity in cancer treatment phases and types—ranging from early aggressive treatments to mild maintenance therapies, as well as the variations in treatment of their basic disease—from chemotherapy alone to multimodal treatment approaches—may affect the generalizability of our results. In addition, our study included a limited number of oncology patients in a small country, therefore, caution should be exercised when interpreting the findings from this study.

## 5 Conclusion

Our study indicates that in Lebanon, COVID-19 was related to treatment changes or delay in oncological children. In addition, a higher proportion of hospitalization, need for intensive care and death were more observed among pediatric cancer patients that tested positive for SARS-CoV-2. Maintaining timely cancer treatment is essential for pediatric oncology patients and should not be interrupted, even in the presence of COVID-19 symptoms, as delays in oncological care can significantly impact patient outcomes. It is crucial that healthcare providers adopt strategies to manage both cancer and COVID-19 concurrently. Vaccination remains essential to provide optimal protection against COVID-19 for this vulnerable population, particularly given the emergence of new SARS-CoV-2 variants (34–38).

## Data Availability

The data analyzed in this study is subject to the following licenses/restrictions: The raw data supporting the conclusions of this article will be made available by the authors on request. Requests to access these datasets should be directed to Adlette Inati, adlette.inati@lau.edu.lb; Peter Noun, peternoun@gmail.com.
